# Trends in antimicrobial resistance of bacterial pathogens in Harare, Zimbabwe, 2012–2017: a secondary dataset analysis

**DOI:** 10.1186/s12879-019-4295-6

**Published:** 2019-08-27

**Authors:** Marvellous Mhondoro, Nqobile Ndlovu, Donewell Bangure, Tsitsi Juru, Notion Tafara Gombe, Gerald Shambira, Peter Nsubuga, Mufuta Tshimanga

**Affiliations:** 1 Department of Community Medicine, University of Zimbabwe, Harare, Zimbabwe; 2 African Society of Laboratory Medicine, Addis Ababa, Ethiopia; 3 Africa Centres for Disease Control and Prevention, Addis Ababa, Ethiopia; 4 Global Health Solutions, Atlanta, GA USA

**Keywords:** Antimicrobial agents, Antibiotic, Resistance

## Abstract

**Background:**

Antimicrobial resistance is one of the most serious public health threats of the twenty-first century. The implementation of AMR surveillance in Zimbabwe is limited. However, data from a private laboratory in Harare revealed increasing resistance rates to common antibiotics like ampicillin (i.e., from 73.9% in 2011 to 74.6% in 2015). The increasing resistance rates indicate that Zimbabwe is affected by AMR. This study was done to determine the magnitude of AMR in Harare and determine the trends of AMR to first-line and to last-resort antibiotics and make recommendations to mitigate the problem.

**Methods:**

A retrospective record review of data collected from the microbiology department at a private laboratory between January 2012 and December 2017 was done. The outcome of interest was the antibiotic susceptibility of bacterial isolates. Microsoft Excel 2016 was used to plot trends from 2012 to 2017 and Epi Info™7 was used for statistical analysis.

**Results:**

A total of 23,432 isolates, of 12 medically important bacteria were analysed. Forty-three percent of the isolates were from urines, 36.7% were from pus swabs and 7% were from blood. The most common pathogen was *Escherichia coli* (43.2%), followed by *Staphylococcus aureus* (15.8%) and the least common was *Neisseria gonorrhoea* (0.2%). Resistance was highest to ampicillin followed by penicillin, both ranging between 70 and 100% over the six years. Statistically significant increases in resistance to commonly used antibiotics were observed in amoxicillin-resistant *E. coli* and *Streptococcus pneumonia* and third generation cephalosporin-resistant *E. coli.* There was an increase in resistance to last-line antibiotics i.e., fluoroquinolone-resistant *Salmonella* spp. and carbapenem-resistant *Pseudomonas aeruginosa* and *Acinetobacter baumannii.* However, methicillin-resistant *S. aureus showed a decreasing trend.*

**Conclusions:**

There is a high burden of drug resistance to common antibiotics in Harare and an emergence of resistance to last-line antibiotics.

## Background

Antimicrobial resistance (AMR) is one of the most serious public health threats of the twenty-first century [1]. Globally, about 700,000 people die due to AMR related illnesses every year. It is estimated that by 2050 these deaths will reach10 million, costing the world US$100 trillion [2]. In 2014, the World Health Organisation (WHO) reported > 25% resistance to penicillin by *Streptococcus pneumoniae* in all its six regions. Five out of the six regions reported > 50% resistance to 3rd generation cephalosporins by *Eschericia coli*. In Africa, AMR surveillance is limited, but the available data suggest up to 100% resistance to beta-lactam antibiotics [3]. WHO reported resistance to last resort antibiotics like vancomycin, 3rd generation cephalosporins, clindamycin and carbapenems by some organisms. Resistance to these last line antibiotics led the WHO to advocate for research and development of more antimicrobials for treatment of these priority organisms in 2017 [4]. In response to this global threat, Africa Centres for Disease Control (CDC) launched the Antimicrobial Resistance Surveillance Network (AMRSNET) which works closely with WHO to strengthen AMR surveillance in Africa [5].

The 2014 WHO global report on AMR showed large gaps in the surveillance of drug resistance in many countries including Zimbabwe [3]. Program focussed AMR surveillance was done in Zimbabwe for malaria, Human Immunodeficiency Virus (HIV) and tuberculosis (TB) but there is a huge knowledge deficit on AMR to common bacterial infections. The overall burden of AMR and its effects on the country’s economy are unknown. Available data for AMR by common bacteria are from studies by academics. A study by Mbanga et al. in 2010 showed high resistance to antibiotics by uro-pathogens e.g., ampicillin (84.5%) and cotrimoxazole (68.5%) [6]. Other studies show AMR in diseases like typhoid and tuberculosis [7, 8]. Drug resistance undermines health delivery programs and threatens the achievement of several United Nations Sustainable Development Goals (SDGs), particularly the targets for good health and wellbeing (i.e., SDG 3) [9].

Data from a reputable technologically advanced Zimbabwean laboratory, accredited by the South African National Accreditation System (SANAS), showed an increasing trend in resistance to the most common antibiotics for example, 73.9% of the isolates were resistant to amoxicillin in 2011 and rose to 74.6% in 2015. The data set is primarily for record keeping of patients’ results. It is used by microbiologists to monitor the antimicrobial susceptibility trends and advise clients (i.e., submitting clinicians) on the effective treatment options.

A retrospective record review on the data was done to describe the trends in antimicrobial resistance by key bacterial organisms and to determine the burden of resistance to last resort antibiotics, to explore reasons for the prevailing trends and provide useful information to clinicians and policy makers.

## Methods

### Aim

The aim of the study was to determine the trends in antibiotic resistance to routinely used antibiotics.

### Study design

A retrospective record review of a data set obtained from microbiology laboratory results was done.

### Study setting

The study was conducted between January and March 2018 using data from a private clinical laboratory in Harare. The laboratory is one of the major diagnostic laboratories in Zimbabwe with more than 50 tests accredited by SANAS in accordance with recognised international standard ISO 15189:2012. The laboratory’s main catchment area is Harare city, with a population of approximately two million people basing on the 2012 census [10]. The accredited microbiology tests include microscopy, culture, identification and antibiotic sensitivity tests. There is an adequate staff complement of proficient scientists with great capacity for quality testing and an information system to store and manage data. The microbiology department receives specimen for microscopy, culture and sensitivity from both private and public health institutions.

### Data source

The data were obtained from a dataset known as “organisms isolated” which stores information from the microbiology department. The data set was created in 2010, and it is embedded in an internationally recognised laboratory information management system called Meditech. Records from 1 January 2012 up to 31 December 2017 were analysed.

The data set consists of at least 65 bacteria species. A purposive sampling of 12 medically important bacteria species was done. They include *Acinetobacter baumannii, Coagulase-negative Staphylococcus, Enterobacter* species*, Enterococcus* species*, Klebsiella pnemoniae, Escherichia coli, Staphylococcus aureus, Pseudomonas aeruginosa, Salmonella species, Campylobacter species, Neisseria gonorrhoea and Streptococcus pneumonia.* Identification of the bacteria was achieved by culturing different specimens onto appropriate media and incubating at 36 °C. The isolated bacteria were identified by Analytical Profile Index (BioMerieux, USA), Microscan system (Beckman Coulter, CA, USA), antisera and biochemical tests (e.g., coagulase and sugars). The laboratory used Kirby-Bauer disk diffusion method on Muller Hinton agar or Microscan system for antibiotic sensitivity testing. A saline suspension of an isolate was standardised using Densicheck plus instrument (BioMerieux, USA) to achieve inoculum density equivalent to McFarlane standard before sensitivity testing. Antibiotic discs were then placed onto the plated agar (not more than 7 discs per plate) and incubated overnight at 36 °C and Clinical and Laboratory Standards Institute guidelines were used for interpreting the susceptibility patterns.

### Data management and analysis

A total of 23,904 patients had at least one organism isolated from the samples. Isolates from the 12 above mentioned organisms were included in the study. For specimens with more than one isolate, only the first recorded isolate was included. All isolates not mentioned above were excluded. If a patient had two identical bacteria isolated less than a month apart, the second one was excluded from analysis. A total of 472 isolates were excluded from the analysis hence the final sample size was 23,432 isolates. The variables analysed included the specimen type (i.e., urines, blood, pus/swabs, penile swabs, seminal fluid, urethral, vaginal, high vaginal, vulval swab, ear, nasal and throat swabs, cerebrospinal fluid and stools), organisms isolated and antibiotics tested (i.e., ampicillin, amoxicillin, augmentin, ciprofloxacin, cotrimoxazole, ceftriaxone, penicillin, gentamicin, carbapenems, vancomycin). The outcome of interest was the antibiotic susceptibility of the isolates. For statistical analysis, isolates were classified as either susceptible or resistant to an antimicrobial and all isolates with intermediate reactions were classified as resistant.

Data quality was checked using completeness of data entries in the Laboratory Information System. Resistance to each antibiotic was analysed separately, using Microsoft Excel 2016 to plot the trends from 2012 to 2017. Simple linear regression was employed to test the significance of antibiotic resistance trends over time. Epi-Info™ 7- (CDC, Atlanta, Georgia) statistical package was used for statistical analysis. The *p*-values < 0.05 were considered statistically significant.

## Results

### Distribution of specimen and bacterial isolates analysed, Harare 2012–17

All records were 100% complete for the variables analysed. Urine was the most common specimen type accounting for 43.4% (10167), followed by pus-swabs and blood which accounted for 36.7% (8588) and 7.4% (1730) respectively. The least common specimen type was cerebrospinal fluid (0.1%). From these specimens, *E. coli* was the most frequent isolate accounting for 43.2% (10130), followed by *S. aureus* and the least frequent was *N. gonorrhoea* which accounted for 0.2% (53) of the isolates (Table 1).

**Table 1:** Distribution of specimen and bacterial isolates analysed, Harare 2012–17

Variable	Category	Frequency (*n* = 23,432)	Percentage (%)
Specimen type	Urine	10,167	43.4
	Pus swab	8588	36.7
	Blood	1730	7.4
	Sputum	1118	4.8
	Ear*	586	2.5
	Nose*	267	1.1
	Throat*	95	0.4
	Penile swab	25	0.1
	Seminal fluid	54	0.2
	Urethral	125	0.5
	Vaginal	117	0.5
	High vaginal swab**	134	0.6
	Vulval swab**	19	0.1
	Stool	384	1.6
	Cerebrospinal fluid	23	0.1
Bacteria species	*E. coli*	10,130	43.2
	*S. aureus*	3703	15.8
	*Coag neg*	2599	11.1
	*staphylococcus*		
	*Enterococcus spp*	1891	8.1
	*P. areuginosa*	1649	7
	*K. pneumoniae*	1299	5.5
	*Enterobacter spp*	834	3.6
	*A.baumanii*	616	2.6
	*Salmonella spp*	384	1.6
	*S.pneumoniae*	137	0.6
	*Campylobacter spp*	137	0.6
	*N. gonorrhoea*	53	0.2

*ENT- ear, nose and throat

**STI- sexually transmitted infections

### Trends of total antimicrobial resistance by common antibiotics, Harare 2012–17

**Fig. 1:**
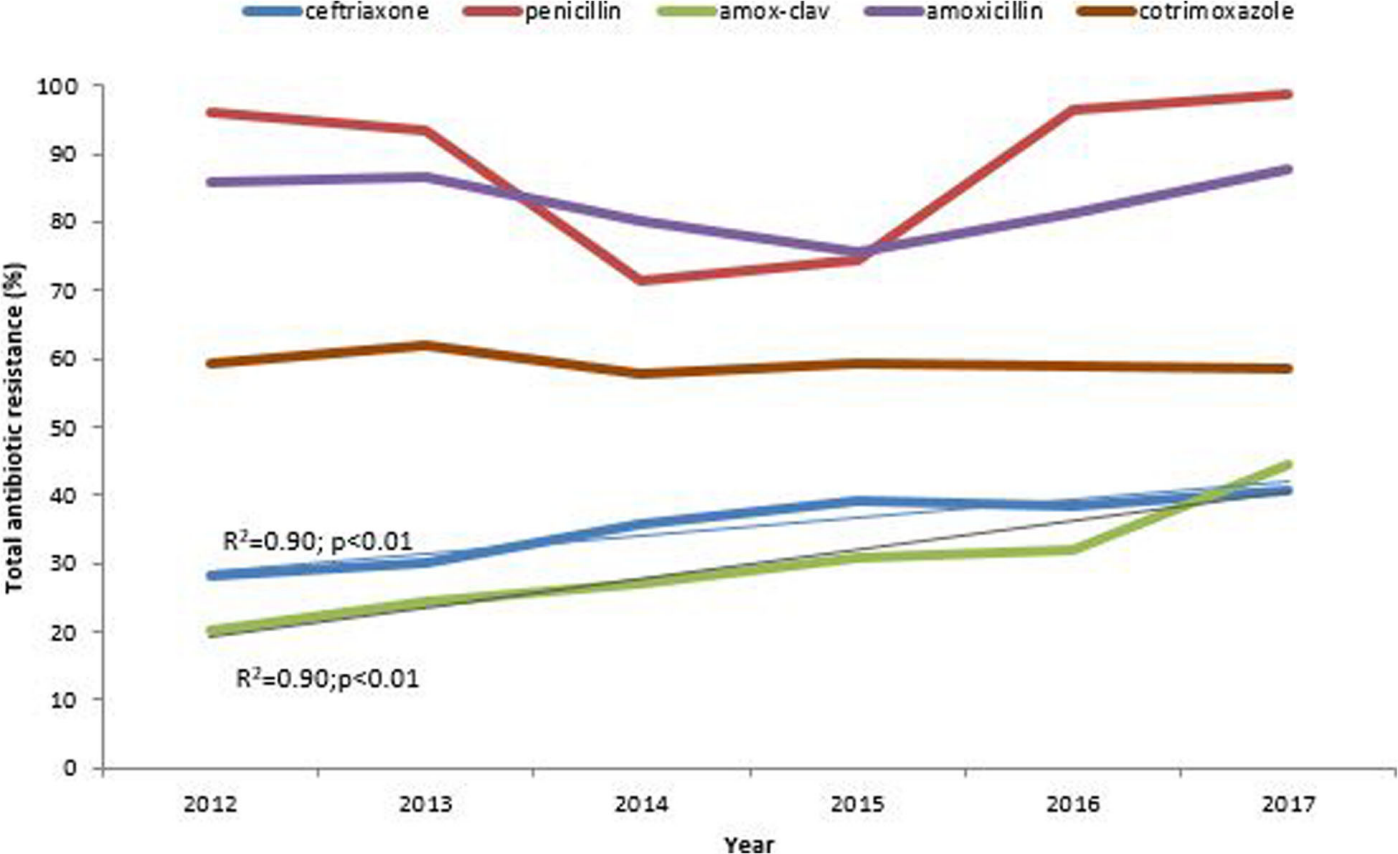
Trends of total antimicrobial resistance by common antibiotics, Harare 2012–17

Ninety-five percent of all bacteria analysed were resistant to penicillin in 2012. The resistance decreased to 71.4% in 2014 and by 2017 it had increased again to 98.7%. Resistance to amoxicillin decreased from 85.9 to 75.8% between 2012 and 2016 before increasing to 87.7% in 2017. Total resistance to cotrimoxazole was constantly high, ranging between 58 and 62% throughout the study period. Over the 6 years there was increasing resistance to ceftriaxone (R^2^ = 0.90; *p* < 0.01) and augmentin (R^2^ = 0.90; *p* < 0.01) (Fig. 1). Resistance to ciprofloxacin (not shown in the figure) showed a slight increase from 28.9% in 2012 to 37.8% in 2017.

### Trends of resistance to common antibiotics by specific organisms, Harare 2012–17

The trends of resistance to different antibiotics by specific organisms between 2012 and 2017 are summarised in Table 2.

**Table 2:** Summary of antibiotic resistance trends, Harare 2012–2017

Antibiotic	Bacteria	Yearly Resistance rates					
		2012	2013	2014	2015	2016	2017
Amoxicillin	*E.coli*	96.2	98.9	88.9	84.1	88.6	97.4
	*S. pneumoniae*	4.4	10.3	11.1	54.6	56.5	56.3
Augmentin	*E.coli*	20.1	23.7	29.7	35.1	36.8	40.2
	*S. pneumoniae*	5.2	15.2	33.3	44.5	71.4	55.6
	*S. aureus*	6.8	8.3	7.3	4.2	3.8	2.0
3rd gen cephalosporins	*E.coli*	20.3	23.5	29.6	33.2	32.2	34.9
	*A.baumannii*	69.1	60.7	61.3	37.5	40.9	29.8
	*P. aeruginosa*	11.4	12.9	12.1	12.1	13.8	9.2
	*Salmonella spp*	7.6	2.7	5.4	10.7	9.4	6.3
	*N. gonorrhoea*	0	0	0	0	0	0
	*S. pneumonia*	0	0	0	0	0	0
Meropenem	*A.baumannii*	1.8	6.9	9.1	4.4	17.0	15.4
	*P.aeruginosa*	1.4	0.5	0.4	0.7	2.9	2.9
	*Ecoli*	0	0	0	0.1	0.2	0
Methicillin	*S. aureus*	6.9	8.5	7.1	4.0	3.7	2.3
Fluoroquinolones	*Salmonella* spp	2.7	0	0	2.6	7.7	6.5
	*Campylobacter* spp	35.3	35.0	46.4	45.0	63.6	40.0

Resistance to amoxicillin was highest among Gram-negative bacteria. *E. coli* had high resistance to amoxicillin throughout the six years i.e., 96.2% in 2012 and decreased to a minimum of 84.1% in 2015 then increased again to 97.4% in 2017 (R^2^ = 0.07; *p* = 0.59). *S. pneumoniae* showed increasing resistance to amoxicillin (i.e., 4.35 to 56.3%) between 2012 and 2017: the trend was statistically significant with R^2^ = 0.83; *p* = 0.01.

**Fig. 2:**
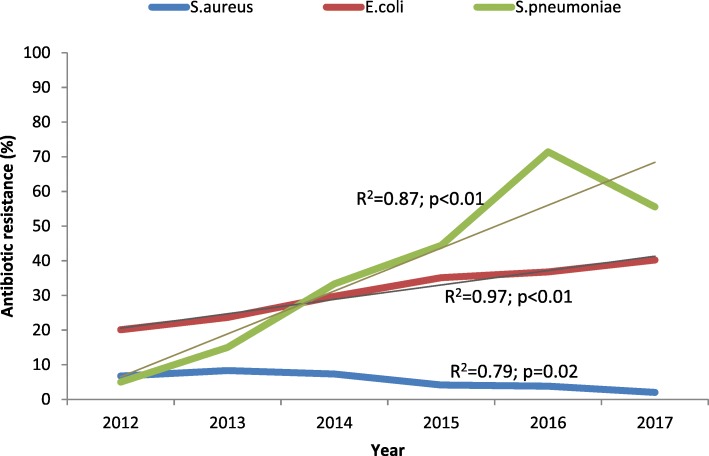
Trends of resistance to augmentin by specific organisms, Harare 2012–17

Resistance to augmentin by *E.coli* was statistically significant i.e., 20.1% in 2012 to 40.2% in 2017 (R^2^ = 0.98; *p* < 0.01)*. S. pneumoniae* also showed increasing resistance to augmentin i.e., from 0% in 2012 to a 71.4% peak in 2016 and then a decrease to 55.5% in 2017 (R^2^ = 0.87; *p* < 0.01)*. S. aureus* showed declining resistance to augmentin from 6.8 to 2.0% between 2012 and 2017 (R^2^ = 0.79; *p* = 0.02) Fig. 2.

### Trends of resistance to cephalosporins by specific organisms, Harare 2012–17

**Fig. 3:**
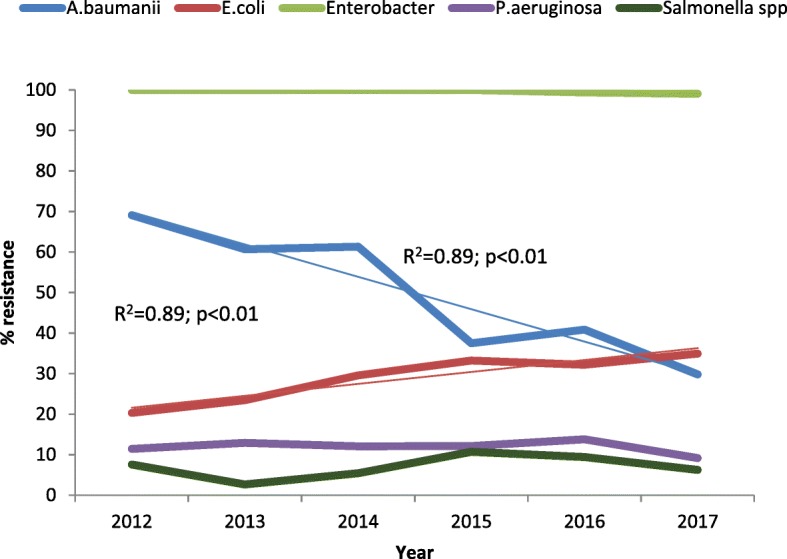
Trends of resistance to cephalosporins by specific organisms, Harare 2012–17

Resistance to cephalosporins by *E. coli* increased from 20.3% in 2012 to 34.9% in 2017 (R^2^ = 0.89; *p* < 0.01) while a decrease was noted in *Acinetobacter baumannii* from 69.1 to 29.8% (R^2^ = 0.89; *p* < 0.01). *Pseudomonas aeruginosa* and *Salmonella* spp. both showed < 15% resistance to 3rd generation cephalosporins (i.e., ceftazidime) between 2012 and 2017 (Fig. 3). *N. gonorrhoea* and *S. pneumoniae* were susceptible to ceftriaxone with 0% resistance over the study period (data not shown).

### Antimicrobial resistance trends WHO priority organisms, Harare 2012–17

**Fig. 4:**
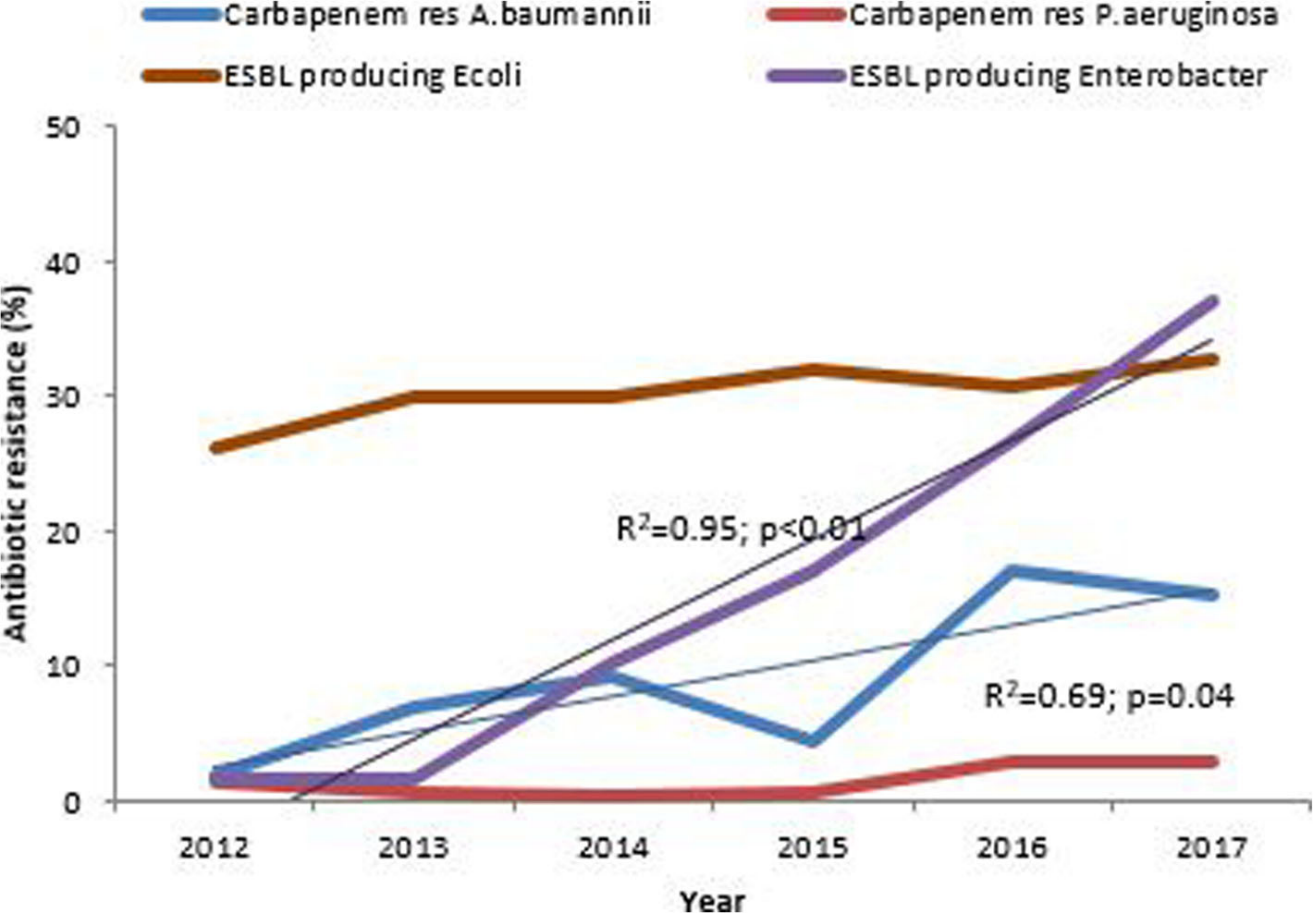
Antimicrobial resistance trends WHO critical priority organisms, Harare 2012–17

There was an emergence of carbapenem resistance over the past six years. In 2012 carbapenem resistance was less than 2% for both *A. baumannii* and *P. aeruginosa* but by 2017 it had increased to 15.4% (R^2^ = 0.69; *p* = 0.04) and 2.9% (R^2^ = 0.573 *p* value = 0.08) respectively. Carbapenem resistant *Enterobacteriaceae* was < 1% between 2012 and 2017 (Fig. 4). There was also an increase in extended spectrum beta-lactamase production in *Enterobacteriaceae* (i.e., *E. coli* and *Enterobacter* spp) over the years. *Enterobacter* spp. showed a statistically significant trend (R^2^ = 0.95; *p* < 0.01).

**Fig. 5:**
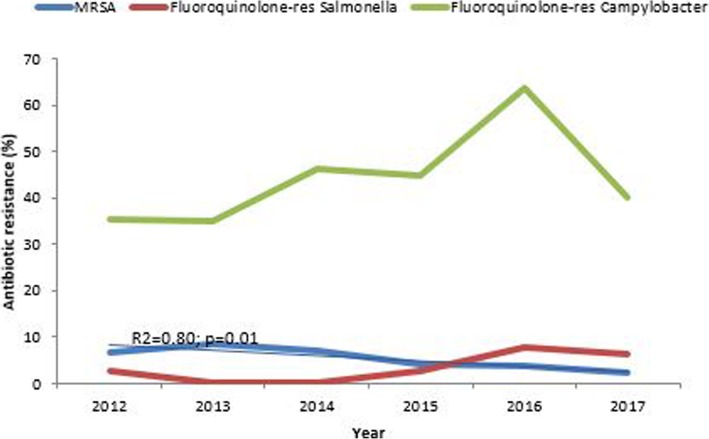
Antimicrobial resistance trends WHO high priority organisms, Harare 2012–17

There was a decrease in methicillin-resistant *S. aureus* (MRSA) from 6.9% in 2012 to 2.3% in 2017 (R^2^ = 0.80; *p* = 0.01). A peak resistance of 8.5% was recorded in 2013. Fluoroquinolone-resistant *Salmonella* species showed an increasing but fluctuating trend over the six years, the lowest resistance rates were recorded between 2013 and 2014 with a peak of 7.7% in 2016 and a slight decline to 6.5% in 2017 (R^2^ = 0.55 *p* = 0.09). Fluoroquinolone resistance was high among *Campylobacter* species; it increased from 35.3% to a peak of 63.6% in 2016 and decreased to 40% in 2017 (R^2^ = 0.30 *p* = 0.27) (Fig. 5).

## Discussion

Several statistically significant changes in antibiotic resistance rates were observed between January 2012 and December 2017. The most important trends were the high resistance rates to commonly used antibiotics and the emerging resistance to last treatment options. According to the AMR situational analysis of 2007, ceftriaxone, benzyl penicillin, cloxacillin, amoxicillin and ciprofloxacin were the most prescribed antibiotics in Zimbabwe [11]. There were high levels of resistance to these antibiotics, consistent with literature which states that the more an antibiotic is used, the more the bacteria can develop resistance against it [12]. Resistance to commonly used antibiotics increases hospital admission time due to longer duration of illness. Also, the patients would require additional tests and would use more expensive drugs for treatment resulting in higher costs being incurred by the patients, their families and the nation’s healthcare system [13]. The increasing resistance rates to last line antibiotics are very worrisome and may lead to the spread of life-threatening infections especially in hospitals [14].

Antimicrobial resistance rates to augmentin and cephalosporins (though much lower than amoxicillin and penicillin) were increasing significantly over time. The increases could be due to a rise in their use as clinicians avoid the highly resisted amoxicillin and penicillin. Increasing resistance to 3rd generation cephalosporins (e.g., ceftriaxone) has also been reported in countries like Korea where it was attributed to the spread of extended spectrum beta-lactamase producing bacteria [15]. Bacteria, especially *Enterobacteriacae* produce enzymes known as beta-lactamases which breakdown the beta-lactam rings in antibiotics like penicillin and amoxicillin, rendering the antibiotics useless against infections [16]. Our study reveals statistically significant increases in the prevalence of extended-spectrum beta-lactamase (ESBL) producing *Enterobacteriaceae*. Magwenzi et al. 2017 reported ESBL carriage in 52% of patients before hospital admission, suggesting endemic ESBL carriage in Harare [17]. Other authors have explained the increasing prevalence of ESBL producing *Enterobacteriaceae* in multiple regions as representing the global expansion of ESBL-producing clones [18].

ESBL producing bacteria are multi-drug resistant (MDR), and this contributes to the high rates of AMR to B-lactams (e.g., penicillin, amoxicillin and ceftriaxone) by Gram-negative bacteria in this study. Treatment of ESBL producing bacterial infections with antibiotics that act as weak labile inducers (e.g., ampicillin and other B-lactams) is clinically inappropriate. Drugs of choice for such infections are usually combinations of 4th generation cephalosporins (e.g., cefepime) plus an aminoglycoside which generally cost more than first-line antibiotics [19].

From the study findings, carbapenem resistance by *Enterobacteriaceae* was very low (i.e., < 1%) throughout the 6 years in this study. The findings are consistent with Magwenzi et al. 2017 who reported a low prevalence of carbapenem-resistant *Enterobacteriaceae* of 1% in Harare [17]. Carbapenems are therefore a treatment of choice for ESBL positive infections. However, the use of carbapenems is costly especially for low-income countries like Zimbabwe which cannot afford extensive usage of these drugs in public health settings. Therefore, measures need to be put in place to decrease the carriage of ESBL producing bacteria in our population.

The results revealed an emergence of carbapenem-resistant *A. baumannii* and *P. aeruginosa.* These organisms are common causes of hospital-acquired infections and are usually multi-drug resistant. This increase in resistance may be due to increased carbapenem use in the treatment of multi-drug resistant infections by specialists in critical care settings, especially in the private sector. Increasing resistance to carbapenems has also been reported in South Africa where possible endemicity was suspected [20, 21]. Carbapenem-resistant organisms are critical priority organisms which are difficult to treat and cause serious infections which may lead to death. The mechanisms of resistance to carbapenems include carbapenemases production, efflux pumps and porin loss [22, 23]. The emergence of carbapenem resistance coincides with a decreased resistance to cephalosporins by *P.aeruginosa* and A. *baumannii* in this study*.* The decrease in resistance to cephalosporins is probably due to a shift in selective pressure as carbapenems have become empirical treatment for *A. baumannii* and *P. aeruginsa* infections in critical care settings.

There was a decrease in methicillin-resistant *Staphylococcus aureus* (MRSA) over the 6 years. The results are consistent with findings from South Africa where MRSA prevalence declined from 36 to 24% between 2006 and 2011 but contrary to findings in Tunisia where it was increasing [24]. However, MRSA rates are much lower in our study than in South Africa. Some explanations potentially account for this observation, the simplest being possible improvements in infection prevention and control practices [25]. Another explanation is a shift in selective pressures away from beta-lactam therapy for staphylococcal infections causing the resistant organisms to be replaced by susceptible ones. Similar trends of declining MRSA prevalence have been reported in USA and Canada [26, 27]. However, the declines in MRSA rates have been attributed to shifts in epidemic strains rather than improved infection control measures.

An increasing trend was seen in fluoroquinolone resistance by *Salmonella* spp. and *Campylobacter* spp. According to the 2011 guidelines for the management of typhoid fever, ciprofloxacin is the drug of choice over the traditional first-line drugs (i.e., chloramphenicol, ampicillin, amoxicillin or cotrimoxazole) [28]. This empirical use of ciprofloxacin is a plausible cause of the increased resistance. Mashe et al. 2016 reported a sharp increase in ciprofloxacin-resistant *S. typhi*. The resistance rates were much lower in the current study probably because all *Salmonella* species included and may have confounded the resistance rates of *S. typhi* [7]. If this increasing resistance trend persists, more effective, appropriate alternative medicines for the treatment of typhoid would have to be used and the treatment guidelines would need to be revised. The increase in fluoroquinolone resistant *Campylobacter* is consistent with Sproston et al. 2018 who found similar findings in the UK [29]. They attributed it to genetic mutations in the quinolone-resistant-determining regions and exchange of drug resistant bacteria between animals and humans through acquisition of qnr genes.

While we can explain some of these trends, there is a lot of genetic dynamism involved in antimicrobial resistance. Also, some resistance genes are acquired from bacteria of a different genus while other resistant strains come from other parts of the world through human migration.

The study had limitations since it was a retrospective study. It could not be ascertained whether the infections were community acquired or nosocomial or whether resistance was primary or secondary. Hence the AMR rates were generalised to the whole study population. Also, the sample comprised approximately 85% specimens from private institutions, therefore, might not adequately represent resistance rates in public health settings.

## Conclusions

From the results of this study, it can be concluded that there is a high burden of AMR to commonly used antibiotics like amoxicillin, penicillin, augmentin, ciprofloxacin and cotrimoxazole. Also, there is an increase in priority organisms which include carbapenem-resistant *A. baumannii*, carbapenem-resistant *P. aeruginosa*, fluoroquinolone-resistant *Salmonella*, and ESBL producing *Enterobacteriaceae*. However, the following organisms are not a major problem in Harare; carbapenem-resistant *Enterobacteriacae*, vancomycin-resistant *Enterococci*, vancomycin-resistant *S. aureus* and 3rd generation cephalosporin-resistant *N. gonorrhoea*.

Development of a robust nationwide surveillance is recommended for monitoring priority organisms so that they are identified and treated before they spread. The antibiotics needed to treat multi-drug resistant infections (e.g., vancomycin and colistin) are too expensive for resource-limited countries, leaving infection prevention as the best strategy for curbing AMR. The use of vaccination for diseases like *S. typhi*, where vaccines are available should be implemented as a prevention method. Further studies should be done, to compare or relate antibiotic prescribing practices and subsequent AMR patterns in Harare. We also recommend comparisons of AMR prevalence in hospital-acquired-infections (HAIs) and community-acquired infections so that interventions may be focussed on appropriately.

## Data Availability

The data that support the findings of this study are available from Lancet Clinical laboratories, but restrictions apply to the availability of these data. Data are however available from the authors upon reasonable request and with Lancet Clinical laboratory.

## Abbreviations

AMR: Antimicrobial resistance
ARMSNET: Antimicrobial Resistance Surveillance Network
CDC: Centres for Disease Control
ESBL: Extended spectrum B-lactamase
HAI: Hospital-acquired infection
MDR: Multi-drug resistant
MRSA: Methicillin-resistant *Staphylococcus aureus*
SANAS: South African National Accreditation System
SDG: Sustainable development goal
WHO: World Health Organisation
